# Association of binge alcohol use with functional outcomes among individuals with COVID-19 infection

**DOI:** 10.1093/alcalc/agae086

**Published:** 2025-01-02

**Authors:** Sebastian T Tong, Michael Gottlieb, Imtiaz Ebna Mannan, Zihan Zheng, Manisha Sinha, Michelle Santangelo, Kristyn Gatling, Efrat Kean, Phillip Watts, Ralph Wang, Juan Carlos Montoy, Ahamed Idris, Samuel MacDonald, Ryan Huebinger, Mandy Hill, Kelli N O’Laughlin, Nicole L Gentile, Jocelyn Dorney, Caitlin Malicki, Joann G Elmore, Kate Diaz Roldan, Gary Chan, Zhenqiu Lin, Robert A Weinstein, Kari A Stephens

**Affiliations:** Department of Family Medicine, University of Washington, 4225 Roosevelt Way NE, Suite 308, Seattle, WA, 98105, United States; Department of Emergency Medicine, Rush University Medical Center, Chicago, IL, 600 S. Paulina St, Chicago, IL, 60612, United States; Center for Outcomes Research and Evaluation (CORE), Section of Cardiovascular Medicine, Yale School of Medicine, 195 Church Street, 5th Floor, New Haven, CT, 06510, United States; Department of Family Medicine, University of Washington, 4225 Roosevelt Way NE, Suite 308, Seattle, WA, 98105, United States; Department of Family Medicine, University of Washington, 4225 Roosevelt Way NE, Suite 308, Seattle, WA, 98105, United States; Department of Emergency Medicine, Rush University Medical Center, Chicago, IL, 600 S. Paulina St, Chicago, IL, 60612, United States; Department of Emergency Medicine, Rush University Medical Center, Chicago, IL, 600 S. Paulina St, Chicago, IL, 60612, United States; Thomas Jefferson University, 1020 Walnut Street, Philadelphia, PA, 19107, United States; Thomas Jefferson University, 1020 Walnut Street, Philadelphia, PA, 19107, United States; University of California, San Francisco, 505 Parnassus Ave, San Francisco, CA, 94143, United States; University of California, San Francisco, 505 Parnassus Ave, San Francisco, CA, 94143, United States; University of Texas Southwestern Medical Center, 5323 Harry Hines Blvd, Dallas, TX, 75390, United States; University of Texas Southwestern Medical Center, 5323 Harry Hines Blvd, Dallas, TX, 75390, United States; University of Texas Health Science Center at Houston, McGovern Medical School, 6431 Fannin St, Houston, TX, 77030, United States; University of Texas Health Science Center at Houston, McGovern Medical School, 6431 Fannin St, Houston, TX, 77030, United States; Department of Emergency Medicine, University of Washington, 1705 NE Pacific Street, Seattle, WA, 98195, United States; Department of Global Health, University of Washington, 3980 15th Ave NE, Seattle, WA, 98105, United States; Department of Family Medicine, University of Washington, 4225 Roosevelt Way NE, Suite 308, Seattle, WA, 98105, United States; Department of Laboratory Medicine and Pathology, University of Washington, 9750 3rd Ave NE, Suite 400, Seattle, WA, 98115, United States; Section of Cardiovascular Medicine, Yale School of Medicine, 333 Cedar Street, New Haven, CT, 06510, United States; Department of Emergency Medicine, Yale School of Medicine, 464 Congress Ave, New Haven, CT, 06519, United States; Division of General Internal Medicine and Health Services Research, David Geffen School of Medicine at the University of California, Los Angeles, 1100 Glendon Avenue, Suite 850, Los Angeles, CA, 90024, United States; Division of General Internal Medicine and Health Services Research, David Geffen School of Medicine at the University of California, Los Angeles, 1100 Glendon Avenue, Suite 850, Los Angeles, CA, 90024, United States; Department of Biostatistics, University of Washington, 1705 NE Pacific St, Seattle, WA, 98195, United States; Center for Outcomes Research and Evaluation (CORE), Section of Cardiovascular Medicine, Yale School of Medicine, 195 Church Street, 5th Floor, New Haven, CT, 06510, United States; Division of Infectious Diseases, Department of Internal Medicine, Rush University Medical Center, 600 S Paulina St, Chicago, IL, 60612, United States; Department of Family Medicine, University of Washington, 4225 Roosevelt Way NE, Suite 308, Seattle, WA, 98105, United States

**Keywords:** alcohol use, binge alcohol, COVID-19 infection

## Abstract

**Aims:**

Alcohol consumption along with negative sequelae from excess alcohol intake increased during the COVID-19 pandemic. We evaluated the association between binge alcohol use and long-term functional outcomes among COVID-19-positive individuals.

**Methods:**

Using a prospective, longitudinal, multisite cohort study design, we evaluated the association between binge alcohol use and mental and physical functional outcomes using Patient-Reported Outcomes Measurement Information System (PROMIS)-29 scores three and six months postinfection. Eligible patients were those who presented with COVID-19-like symptoms, tested positive for COVID-19, and completed a three-month survey. Binge drinking was identified at the time of infection using the Tobacco, Alcohol, Prescription medication and other Substance use screener. Generalized estimating equation models, adjusted for demographic characteristics, social determinants of health, substance use, comorbidities, and COVID-19 vaccine status, were used to assess the association between binge alcohol use and mental and physical functional outcomes.

**Results:**

Of 3529 individuals, 23.7% screened positive for binge drinking. At three months, prior self-reported binge drinking was associated with differences in physical function [estimate: 1.08; 95% confidence interval (CI) 0.44, 1.71], pain interference (estimate: −0.86; 95% CI −1.57, −0.15), and physical health (estimate: 1.09; 95% CI 0.43, 1.75). At six months, no associations were found between binge drinking and outcomes.

**Conclusions:**

Binge alcohol use before COVID-19 infection was associated with statistically significant but clinically irrelevant improvements in function at three months, which were not sustained at six months. Postinfectious and postpandemic stressors may have played a larger impact on functional outcomes than binge alcohol use. A higher frequency of binge drinking and its association with functional outcomes, particularly among individuals with COVID-19 warrants further study.

## Teaser

**Short Summary:** Using a prospective, longitudinal cohort study design, we evaluated the association between binge alcohol use and long-term functional outcomes among COVID-19-positive individuals. Binge alcohol use before COVID-19 infection was associated with statistically significant but clinically irrelevant improvements in function at 3 months, which were not sustained at six months.

## Introduction

Alcohol use increased substantially during the COVID-19 pandemic. Alcohol sales increased a reported 300% (Bantounou 2023), with concomitant rises in alcohol-related hospitalizations (Sharma et al. 2021) and mortality (White et al. 2022). The number of people engaging in binge drinking weekly during the pandemic increased significantly (Barbosa et al. 2021). Pandemic-related psychological distress and anxiety were associated with greater numbers of binge drinking episodes (Rodriguez et al. 2020), and increased heavy episodic drinking frequency was associated with mental health symptoms.

The pandemic did not increase alcohol consumption across the board; increases were associated with specific person-level and contextual risk factors such as financial stress, unemployment, race, ethnicity, and gender (Acuff et al. 2022). Compared to white participants**,** non-White participants had greater increases in alcohol consumption (Barbosa et al. 2021). A greater number of nonwhite participants (13.8%) reported engaging in extreme binge drinking than did white participants (5.9%). In addition, the rate of increase in alcohol consumption in females was substantially higher than in males during the pandemic.

Several studies have attempted to assess the harms of increased alcohol consumption (Bantounou 2023). Overall, alcohol-related deaths increased by 26% between 2019 and 2020 (White et al. 2022). A 34% increase in alcohol withdrawal hospitalizations was found in 2020 compared to 2019 (Sharma et al. 2021). Increased alcohol consumption was also linked to an increase in alcohol-associated liver disease; from 2019 to 2020, alcohol-associated liver disease mortality increased by 21% in males and 27% in females (Deutsch-Link et al. 2022). Alcohol-associated hepatitis admissions increased by 50% in 2020 from 2016 to 2019 (Gonzalez et al. 2022). Other research has found that people with alcohol use disorder had more severe COVID-19 outcomes (Baillargeon et al. 2021).

While disease-related markers are important, there is a growing movement to understand function in the context of whole-person well-being. A recent National Academies of Sciences, Engineering and Medicine report recommended a shift from a focus on disease-specific outcomes to overall well-being and patient-centered outcomes (*Committee on Transforming Health Care to Create Whole Health: Strategies to Assess, Scale, and Spread the Whole Person Approach to Health, Board on Health Care Services, Health and Medicine Division, National Academies of Sciences, Engineering, and Medicine*  2023). The Diagnostic Statistical Manual 5th edition frames the diagnoses of substance use disorders around how use of substances affects daily life and function rather than on disease-specific measures or biomarkers (https://www.niaaa.nih.gov/publications/brochures-and-fact-sheets/alcohol-use-disorder-comparison-between-dsm  n.d.). Federal funding agencies are also increasing attention on patient-reported outcomes, many of which include function (Blanco et al. 2022). Our study aimed to elucidate whether functional status after COVID-19 infection differed between people who did vs did not binge drink.

## Materials and Methods

This was a secondary analysis of the Innovative Support for Patients with SARS-CoV-2 Registry (INSPIRE), a prospective, multicenter, longitudinal cohort study in the USA. Details about the study design and outcomes can be found here (O’Laughlin et al. 2022). Briefly, patients with COVID-19-like symptoms who received a Food and Drug Administration (FDA)-approved SARS-CoV-2 test were enrolled between December 2020 and August 2022 with follow-up surveys through February 2023. Participants who tested positive and completed a baseline and a 3-month survey including questions about their functioning and substance use were analyzed; 6-month surveys completed by any participants were included as well. During the baseline survey, participants were asked about binge drinking using the Tobacco, Alcohol, Prescription medication and other Substance use (TAPS-1) screener, which queries about the past 12 months (Gryczynski et al. 2017). The definition of binge drinking was drawn from the National Institute on Alcohol Abuse and Alcoholism, which corresponds to more than or equal to five alcoholic beverages on the same occasion for males and more than or equal to four beverages drinks for females (https://www.niaaa.nih.gov/alcohol-health/overview-alcohol-consumption/moderate-binge-drinking  n.d.). The PROMIS-29 questionnaire was used as the primary outcome with measures including physical health and function, social function, anxiety, depression, fatigue, sleep disturbance, pain interference, pain intensity, and mental health (Hays et al. 2018). In addition, cognitive function was measured using PROMIS SF-CF 8a. All measures, except pain intensity, were transformed into *T*-scores.

As per the TAPS-1 screener validation (Gryczynski et al. 2017), daily, weekly, and monthly binge drinking were grouped into the binge drinking group; less than monthly and no binge drinking were grouped into the nonbinge drinking group. We analyzed demographic and clinical characteristics across groups with varying binge drinking frequencies using descriptive statistics and chi-square tests. Generalized estimating equation models were applied to examine the association between binge drinking frequency categories and each of the functional outcomes among COVID-19-positive participants, considering interactions between baseline binge drinking status and time point. Each model was adjusted for age, gender, ethnicity, race, education level, annual family income, substance use, tobacco use in the past 12 months, comorbidities, and the receipt of COVID-19 vaccination. Marginal estimates were reported at each time point to compare the differences between binge drinkers and nonbinge drinkers. No adjustments were made for multiple comparisons due to the exploratory nature of the study. Analyses were conducted using SAS 9.4. This study was approved by the institutional review boards at all study sites.

## Results

Of 3529 individuals who completed baseline and 3-month follow-up surveys, 1.3% reported daily, 9.4% weekly, 13.1% monthly, and 23.0% reported less than monthly binge drinking in the baseline survey. For patients who screened positive for binge drinking, younger ages, male gender, white race, and 4-year college degrees were more common (Table 1).

**Table 1:** Characteristics of the underlying COVID-positive cohort stratified by baseline binge drinking categories (*N* = 3529)

	**Binge drinking**			
	**Binge (*N* = 838)**	**Nonbinge (*N* = 2691)**	**Total (*N* = 3529)**	***P*-value**
	***N* (%)**	***N* (%)**	***N* (%)**	
**Age (at enrollment)**				
18–34	463 (55.52)	970 (36.47)	1433 (41.01)	<.001^***^
35–49	244 (29.26)	889 (33.42)	1133 (32.43)	
50–64	100 (11.99)	549 (20.64)	649 (18.57)	
65+	27 (3.24)	252 (9.47)	279 (7.99)	
Missing	3	22	25	
**Gender**				
Male	295 (35.89)	797 (30.61)	1092 (31.87)	0.007^**^
Female	513 (62.41)	1776 (68.20)	2289 (66.81)	
Transgender/nonbinary other	14 (1.70)	31 (1.19)	45 (1.31)	
Missing	15	78	93	
**Race**				
White	612 (74.36)	1821 (69.88)	2433 (70.95)	.041^^*^^
Black	52 (6.32)	179 (6.87)	231 (6.74)	
Asian	86 (1.45)	366 (14.04)	452 (13.18)	
Other/Multiple	73 (8.87)	240 (9.21)	313 (9.13)	
Missing	14	76	90	
**Ethnicity**				
Non-Hispanic	724 (87.55)	2268 (86.24)	2992 (86.55)	.34
Hispanic	103 (12.45)	362 (13.76)	465 (13.45)	
Missing	10	52	62	
**Education**				
Less than high school	7 (0.85)	21 (0.80)	28 (0.81)	<.001^***^
High school graduate	58 (7.04)	139 (5.30)	197 (5.72)	
Some college	117 (14.20)	334 (12.74)	451 (13.09)	
2-year degree	46 (5.58)	183 (6.98)	229 (6.65)	
4-year degree	334 (40.53)	844 (32.20)	1178 (34.19)	
More than 4 years	262 (31.80)	1100 (41.97)	1362 (39.54)	
Missing	13	61	74	
**Family income (prepandemic)**				
<10 000	47 (5.62)	119 (4.44)	166 (4.72)	.029^^*^^
10 000–34 999	89 (10.63)	257 (9.58)	346 (9.83)	
35 000–49 999	83 (9.92)	244 (9.10)	327 (9.29)	
50 000–74 999	120 (14.34)	329 (12.27)	449 (12.76)	
75 000+	461 (55.08)	1546 (57.64)	2007 (57.03)	
Unknown	37 (4.42)	187 (6.97)	224 (6.37)	
Missing	0	0	0	
**Tobacco use at baseline**				
Daily or nearly daily	68 (8.12)	104 (3.88)	172 (4.89)	<.001^***^
Weekly	34 (4.06)	19 (0.71)	53 (1.51)	
Less than monthly	101 (12.07)	83 (3.10)	184 (5.23)	
Monthly	38 (4.54)	11 (0.41)	49 (1.39)	
Not at all	596 (71.21)	2463 (91.90)	3059 (86.98)	
Missing	0	2	2	
**Prescription drug misuse at baseline**				
Daily or nearly daily	11 (1.32)	28 (1.04)	39 (1.11)	<.001^***^
Weekly	8 (0.96)	6 (0.22)	14 (0.40)	
Less than monthly	34 (4.07)	58 (2.16)	92 (2.62)	
Monthly	13 (1.56)	11 (0.41)	24 (0.68)	
Not at all	770 (92.11)	2578 (96.16)	3348 (95.19)	
Missing	1	1	2	
**Marijuana use at baseline**				
Daily or nearly daily	71 (8.50)	94 (3.51)	165 (4.69)	<.001^***^
Weekly	78 (9.34)	91 (3.39)	169 (4.81)	
Less than monthly	147 (17.60)	262 (9.77)	409 (11.63)	
Monthly	79 (9.46)	66 (2.46)	145 (4.12)	
Not at all	460 (55.09)	2168 (80.87)	2628 (74.74)	
Missing	2	1	3	
**Illicit drug use at baseline**				
Daily or nearly daily	2 (0.24)	8 (0.30)	10 (0.28)	<.001^***^
Weekly	3 (0.36)	4 (0.15)	7 (0.20)	
Less than monthly	90 (10.75)	67 (2.50)	157 (4.46)	
Monthly	8 (0.96)	4 (0.15)	12 (0.34)	
Not at all	734 (87.69)	2597 (96.90)	3331 (94.71)	
Missing	0	2	2	
**Comorbidities**				
Asthma	83 (1.00)	322 (12.22)	405 (11.69)	.08
Hypertension	88 (10.60)	365 (13.85)	453 (13.07)	.015^*^
Diabetes	27 (3.25)	130 (4.93)	157 (4.53)	.042^^*^^
Obesity	196 (23.61)	721 (27.36)	917 (26.46)	.033^^*^^
Emphysema/COPD	4 (0.48)	20 (0.76)	24 (0.69)	.4
Heart conditions	12 (1.45)	63 (2.39)	75 (2.16)	.1
Kidney disease	1 (0.12)	43 (1.63)	44 (1.27)	<.001^^***^^
Liver disease	3 (0.36)	18 (0.68)	21 (0.61)	.3
**COVID-19 vaccination status at baseline**				
No	169 (24.93)	487 (23.27)	656 (23.67)	.38
Yes	509 (75.07)	1606 (76.73)	2115 (76.33)	
Missing	159	589	748	

Note: The table includes participants who tested positive at the index COVID-19 test and completed a baseline and a 3-month survey. Binge drinking is defined as more than or equal to five alcoholic beverages on the same occasion for males and more than or equal to four beverage drinks for females. We grouped those who reported daily, weekly, or monthly together in the binge drinking category and those who reported less than monthly or never together into the nonbinge drinking category. *N* = 2 participants were missing for the binge drinking variable. For the comorbidity questions, individuals were asked to indicate whether or not they had any of the listed comorbidities in a binary fashion. This was only asked on 3-month survey starting 14 April 2021 and has missing data for binge drinkers (*n* = 7) and nonbinge drinkers (*n* = 47). *P*-values are from the bivariate chi-square tests conducted to determine association between binge drinking and the respective row variable. COVID-19 vaccination indicates participants with at least one dose before the index COVID-19 test.

^*^ *P*-value <.05.

^**^ *P*-value <.01.

^***^ *P*-value <.001.

**Figure 1:**
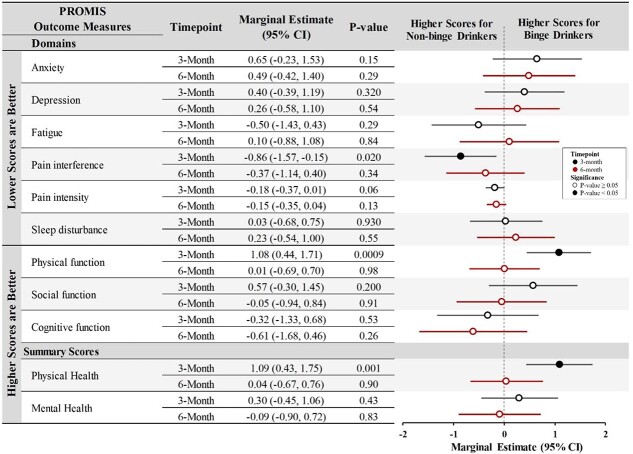
Marginal estimates from GEE analysis for functional outcomes for binge alcohol drinkers in reference to nonbinge drinkers at 3 and 6 months for the cohort who tested positive at the index COVID-19 test. Note: Marginal estimates from GEE analysis for functional outcomes for binge alcohol drinkers in reference to nonbinge drinkers are calculated. Three-month estimates include participants with baseline and 3-month surveys. Six-month estimates include a subset of these participants, for which 6-month surveys were available. PROMIS outcome measures include *T*-scores from PROMIS-29 domains: anxiety, depression, fatigue, pain interference, sleep disturbance, physical function, and social function. An additional measure for pain intensity (scaled 0–10) is included. Cognitive function is measured using PROMIS SF-CF 8a. Physical and mental health summary scores are also analyzed as outcome measures.

According to the marginal estimates (Fig. 1), at 3 months, those who screened positive for baseline binge alcohol use were associated with better physical function (estimate : 1.08, 95% CI: [0.44, 1.71]), less pain interference (estimate: −0.86, 95% CI: [−1.57, −0.15]), and better physical health overall (estimate : 1.09, 95% CI: [0.43, 1.75]). Other PROMIS-29 subscales (including social function, anxiety, depression, fatigue, sleep disturbance, pain intensity, and composite mental health summary score) had no significant association with binge drinking. At 6 months, no significant associations with reported binge drinking at baseline were found with any functional outcomes.

## Discussion

Our study describes the functional outcomes over 6 months for a large cohort of COVID-19-positive individuals, of whom 23.7% screened positive for binge drinking at baseline. Unlike other studies that focused on alcohol use and alcohol-related health care utilization, our study focused on the patient-reported, validated, functional outcomes in the months following COVID-19 infection of those reporting binge alcohol use at baseline. Using baseline, 3-, and 6-month surveys, we found marginally improved functional outcomes for binge drinkers at 3 months, suggesting that binge drinking at the time of COVID-19 infection may not be associated with demonstrable differences in function when followed longitudinally.

Several potential reasons exist for the lack of association between baseline binge drinking and long-term functional outcomes. First, binge drinking during the pandemic could potentially have been transient and not result in differences in long-term functional outcomes. Second, postinfectious and postpandemic stressors may have played a larger impact on functional outcomes than binge drinking. Within the context of other stressors, alcohol binge use may be a symptom of other physiologic and social stressors rather than uniquely a predictor of poor functional outcomes. This suggests that interventions targeting alcohol use need to also address the contextual factors that lead to increased alcohol consumption. Finally, at least one study has suggested a dose-dependent relationship between alcohol consumption and immune function, and this dose relationship is not fully described within the context of the TAPS-1 tool (Barr et al. 2016). Further studies are needed examining binge use in the context of other larger stressors and how we might best address binge use within this larger context of whole-person health. Better understanding the complex interplay of psychological, immunological, social, and temporal factors that contribute to the relationship between alcohol use and function can help with developing effective interventions to address unhealthy alcohol use.

There are several limitations to our study. First, underreporting of binge alcohol use is common, although this is mitigated by the use of the standardized screening instrument. Second, nonbinge alcohol use was not captured in the survey and thus could not be controlled for as a variable. Third, alcohol use was only captured at baseline and was not collected at the 3-month and 6-month follow-up, so findings may not generalize to those who continued to binge drink post-COVID-19 infection.

## Conclusion

Binge alcohol use before COVID-19 infection was associated with slightly better physical and less pain-related function at 3 months, which was not sustained at 6 months. Post-infectious and pandemic stressors that may have played a larger impact on functional outcomes than binge alcohol use must be explored.

## Supplementary Material

Appendix_INSPIRE_Group_Acknowledgements_List_06_20_24_CLEAN_agae086

## Data Availability

Study data are owned and managed directly by the grant recipient (Rush University) and the funder (Centers for Disease Control and Prevention). The data underlying the results presented in the study are from the INSPIRE Registry. The coordinating center, Rush University, can be contacted via email at inspirepub@rush.edu to request information related to confidential data.

## Statement of adherence to preprint policy

We have not published this article in a preprint server and we are in adherence with the journal’s policy.

## References

[ref1] Acuff SF, Strickland JC, Tucker JA. et al. Changes in alcohol use during COVID-19 and associations with contextual and individual difference variables: A systematic review and meta-analysis Psychol Addict Behav. 2022;36:1–19. 10.1037/adb0000796. 34807630 PMC8831454

[ref2] Baillargeon J, Polychronopoulou E, Kuo YF. et al. The impact of substance use disorder on COVID-19 outcomes Psychiatr Serv. 2021;72:578–81. 10.1176/appi.ps.202000534. 33138712 PMC8089118

[ref3] Bantounou MA . A narrative review of the use of alcohol during the Covid-19 pandemic; effects and implications J Addict Dis. 2023;41:30–40. 10.1080/10550887.2022.2058852. 35373718

[ref4] Barbosa C, Cowell AJ, Dowd WN. Alcohol consumption in response to the COVID-19 pandemic in the USA J Addict Med. 2021;15:341–4. 10.1097/ADM.0000000000000767. 33105169 PMC8327759

[ref5] Barr T, Helms C, Grant K. et al. Opposing effects of alcohol on the immune system Prog Neuro-Psychopharmacol Biol Psychiatry. 2016;65:242–51. 10.1016/j.pnpbp.2015.09.001. PMC4911891 26375241

[ref6] Blanco C, Kato EU, Aklin WM. et al. Research to move policy — Using evidence to advance health equity for substance use disorders N Engl J Med. 2022;386:2253–5. 10.1056/NEJMp2202740. 35687034

[ref7] Krist AH, South-Paul J, Meisnere M (eds.). Committee on transforming health care to create whole health: Strategies to assess, scale, and spread the whole person approach to health, board on health care services, health and medicine division, National Academies of sciences, engineering, and medicine. In: *Achieving Whole Health: A New Approach for Veterans and the Nation*. Washington, DC: National Academies Press, 2023, 26854. 10.17226/26854.

[ref8] Deutsch-Link S, Curtis B, Singal AK. Covid-19 and alcohol associated liver disease Dig Liver Dis. 2022;54:1459–68. 10.1016/j.dld.2022.07.007. 35933291 PMC9349236

[ref9] Gonzalez HC, Zhou Y, Nimri FM. et al. Alcohol-related hepatitis admissions increased 50% in the first months of the COVID-19 pandemic in the USA Liver Int. 2022;42:762–4. 10.1111/liv.15172. 35094494

[ref10] Gryczynski J, McNeely J, Wu LT. et al. Validation of the TAPS-1: A four-item screening tool to identify unhealthy substance use in primary care J Gen Intern Med. 2017;32:990–6. 10.1007/s11606-017-4079-x. 28550609 PMC5570743

[ref11] Hays RD, Spritzer KL, Schalet BD. et al. PROMIS®-29 v2.0 profile physical and mental health summary scores Qual Life Res. 2018;27:1885–91. 10.1007/s11136-018-1842-3. 29569016 PMC5999556

[ref12] Drinking Levels Defined | National Institute on Alcohol Abuse and Alcoholism (NIAAA). Accessed May 1, 2024. https://www.niaaa.nih.gov/alcohol-health/overview-alcohol-consumption/moderate-binge-drinking

[ref13] National Institute on Alcohol Abuse and Alcoholism. Alcohol Use Disorder: A Comparison Between DSM–IV and DSM–5 2024. Available from: https://www.niaaa.nih.gov/publications/brochures-and-fact-sheets/alcohol-use-disorder-comparison-between-dsm

[ref14] O’Laughlin KN, Thompson M, Hota B, Gottlieb M, Plumb ID, Chang AM, Wisk LE, Hall AJ, Wang RC, Spatz ES, Stephens KA, Huebinger RM, McDonald SA, Venkatesh A, Gentile N, Slovis BH, Hill M, Saydah S, Idris AH, Rodriguez R, Krumholz HM, Elmore JG, Weinstein RA, Nichol G, for the INSPIRE Investigators Study protocol for the innovative support for patients with SARS-COV-2 infections registry (INSPIRE): A longitudinal study of the medium and long-term sequelae of SARS-CoV-2 infection. Chi G, ed. PLoS One. 2022;17:e0264260. doi:10.1371/journal.pone.0264260 35239680 PMC8893622

[ref15] Rodriguez LM, Litt DM, Stewart SH. Drinking to cope with the pandemic: The unique associations of COVID-19-related perceived threat and psychological distress to drinking behaviors in American men and women Addict Behav. 2020;110:106532. 10.1016/j.addbeh.2020.106532. 32652385 PMC7320671

[ref16] Sharma RA, Subedi K, Gbadebo BM. et al. Alcohol withdrawal rates in hospitalized patients during the COVID-19 pandemic JAMA Netw Open. 2021;4:e210422. 10.1001/jamanetworkopen.2021.0422. 33656526 PMC7930922

[ref17] White AM, Castle IJP, Powell PA. et al. Alcohol-related deaths during the COVID-19 pandemic JAMA. 2022;327:1704. 10.1001/jama.2022.4308. 35302593 PMC8933830

