# Correction to: Association of binge alcohol use with functional outcomes among individuals with COVID-19 infection

**DOI:** 10.1093/alcalc/agaf008

**Published:** 2025-02-26

This is a correction to: Sebastian T Tong *et al*., Association of binge alcohol use with functional outcomes among individuals with COVID-19 infection, *Alcohol and Alcoholism*, Volume 60, Issue 1, January 2025, https://doi.org/10.1093/alcalc/agae086

Supplementary information detailing members and roles in the INSPIRE study team was omitted from the version originally published and has been added.

